# Clinical features and risk factors of plastic bronchitis caused by *Mycoplasma pneumoniae* pneumonia in children

**DOI:** 10.1186/s12890-023-02766-0

**Published:** 2023-11-23

**Authors:** Lei Yang, Yuyan Zhang, Changqing Shen, Zhouhua Lu, Tongshu Hou, Fenghai Niu, Yuzhong Wang, Jun Ning, Ruihan Liu

**Affiliations:** 1 Affiliated Hospital of Jining Medical University, Jining Medical University, Jining, Shandong 272000 China; 2 Postdoctoral Mobile Station of Shandong University of Traditional Chinese Medicine, Jinan, Shandong 250399 China; 3 The Second Clinical Medical College, Binzhou Medical University, Yantai, Shandong 264100 China

**Keywords:** *Mycoplasma pneumoniae* Pneumonia, Plastic bronchitis, Children, Risk factors, Clinical features

## Abstract

**Background:**

We analyzed the clinical characteristics of children with plastic bronchitis (PB) caused by *Mycoplasma pneumoniae* (MP) and explored its risk factors.

**Methods:**

We prospectively analyzed clinical data of children with MP pneumonia (MPP) treated with fiberoptic bronchoscopy (FB). Patients were classified into a PB and non-PB group. General information, clinical manifestations, laboratory tests, results of computed tomography scan, and FB findings were compared between groups. We conducted statistical analysis of risk factors for developing PB.

**Results:**

Of 1169 children who had MPP and were treated with FB, 133 and 1036 were in the PB and non-PB groups, respectively. There were no significant differences in sex, age, and incident season between groups (*P* > 0.05). The number of children in the PB group decreased during the COVID-19 pandemic. Compared with children in the non-PB group, those in the PB group had longer duration of hospitalization, increased levels of neutrophil (N), C-reactive protein (CRP), procalcitonin (PCT), D-dimer, lactate dehydrogenase (LDH), alanine transaminase (ALT) and aspartate transaminase (AST); lower levels of lymphocyte (L) and platelet (PLT); and higher incidence of lack of appetite, decreased breath sounds, single lobar infiltrate, pleural effusion, pericardial effusion, mucosal erosion and/or necrosis, and bronchial embolization. L levels and pleural effusion were identified as risk factors in multivariate logistic regression.

**Conclusions:**

Children with PB caused by MPP had a strong and local inflammatory response. L levels and pleural effusion were independent risk factors of PB with MPP in children. Our findings will help clinicians identify potential PB in pediatric patients for early and effective intervention.

## Background

*Mycoplasma pneumoniae* (MP) is a common lower respiratory tract pathogen that causes MP pneumonia (MPP) in children. MPP is a major cause of community-acquired pneumonia (CAP), accounting for 10−40% of hospitalizations among children [1–11]. MPP is considered self-limiting and benign, but in recent years, several studies have shown that MPP can cause plastic bronchitis (PB) in children [12–16]. PB is an acute and critical pulmonary disease characterized by the formation of bronchial casts (BCs), which can partially or completely obstruct the tracheobronchial tree [17–19]. PB caused by infection in pediatric patients usually presents with productive cough, progressive dyspnea, repeated high fever, or pleuritic chest pain [17, 18]. Fiberoptic bronchoscopy (FB) and bronchoalveolar lavage (BAL) have high efficacy in the diagnosis and treatment of PB [12, 19]. In this prospective study, we analyzed the data of 1169 children with MPP who underwent FB. We aimed to explore the clinical features and risk factors of PB in children with MPP.

## Methods

### Study population

**Fig. 1:**
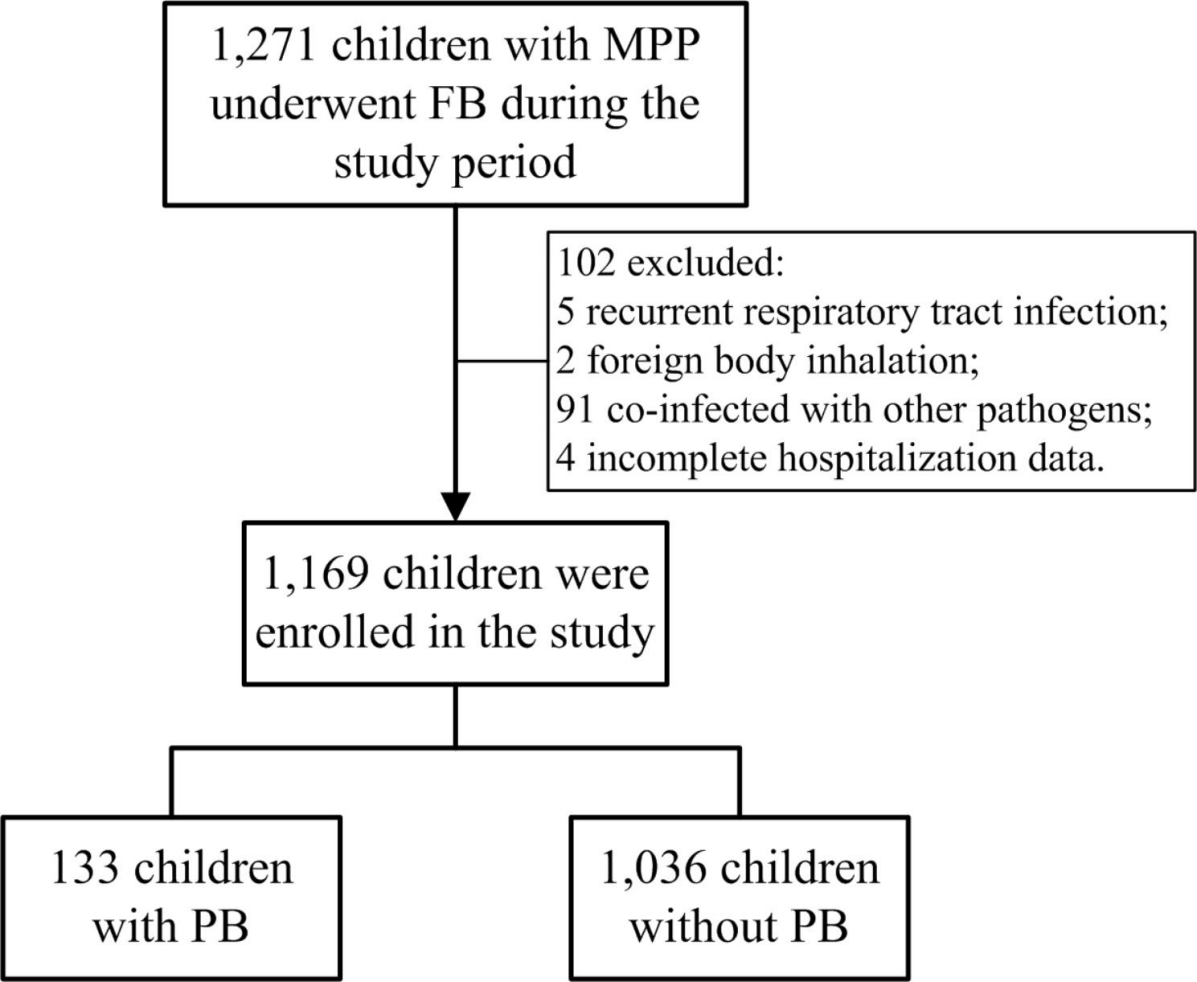
Flowchart of the study population

This study was approved by the Ethics Committee of the Affiliated Hospital of Jining Medical University (No. 2018C076). Children with MPP who were admitted to the department of pediatrics in the Affiliated Hospital of Jining Medical University from February 2019 to January 2020 and from August 2021 to July 2022 and were treated with FB were selected as study participants. The patients were divided into a PB group and a non-PB group, according to whether there was a plastic shape under FB. Inclusion criteria were: (1) hospitalized patients between 1 month and 14 years old; (2) symptoms and signs indicative of CAP, including fever, cough, abnormal lung auscultation, and new infiltration on chest radiograph; (3) positive laboratory results for MP, including an MP immunoglobulin M titer ≥ 1:160 or four-fold rising titer in acute and convalescent serum specimens; positive results for MP polymerase chain reaction tests in BAL fluid [20, 21]; (4) the patient’s condition met the diagnostic criteria of PB: plastic foreign body removed on FB; (5) informed consent was signed for FB and BAL; and (6) complete hospitalization data were available. The exclusion criteria were as follows: (1) previous recurrent respiratory tract infection, asthma, chronic lung disease, operation after cardiac disease, severe blood system disease, or immune deficiency disease; (2) foreign body inhalation; (3) patients currently recovering from MPP; (4) patients co-infected with other pathogens or tuberculosis; and (5) incomplete hospitalization data. Flowchart of the study population see Fig. 1.

### Data collection

The clinical data of children with MPP were collected and mainly included the following: (1) general information: sex, age, etiological diagnostic methods and admission time (incident season); (2) clinical manifestations: time from illness onset to admission, duration of hospitalization, duration of fever and cough (time from the onset of fever and cough to the date of hospitalization), fatigue, dyspnea, wheezing, and other symptoms; decreased breath sounds, rales, rhonchi, hypoxia, and other signs; (3) laboratory tests: routine blood tests, inflammatory markers, and blood biochemistry; (4) computed tomography (CT) scan results; and (5) FB findings and histopathological examination.

### Statistical analysis

All statistical analyses were performed using SAS software, version 9.4 (SAS Institute Inc., Cary, NC, USA). Enumeration data were expressed as percentage (%), measurement data with a normal distribution were described as mean ± standard deviation, and measurement data with a non-normal distribution were described as median (interquartile range). Fisher’s exact test was used for categorical data. The Kruskal–Wallis H test was used for continuous data. Logistic regression analysis was used to examine risk factors that were significant in the multivariate analysis. *P* < 0.05 was considered statistically significant.

## Results

### General information

A total of 1169 MPP children were eligible for inclusion. The PB group accounted for 133 (11.38%) cases and the non-PB group for 1036 (88.62%) cases. Male children comprised 53.38% (71 of 133) of the PB group and 54.15% (561 of 1036) of the non-PB group. The mean age of patients in the PB and non-PB group was 80.40 ± 29.85 months and 74.08 ± 30.68 months, respectively. Three methods were used to detect the pathogen, including single elevated IgM, seroconversion and PCR in BAL. The PCR in BAL was used to confirm MP infection. By using single elevated IgM as a diagnostic method, 119 children from the PB group (accounting for 93.23%, 119 of 133), and 862 children from the non-PB group (accounting for 83.20%, 862 of 1036) were found to have MP infection. The diagnostic method of seroconversion revealed that 120 children from the PB group (accounting for 90.23%, 120 of 133), and 859 children from the non-PB group (accounting for 82.92%, 859 of 1036) were infected with MP. The proportion of children hospitalized in autumn was significantly higher than that in other seasons. There were no significant differences in sex, age, etiological diagnostic methods and incident season between the two groups (*P* > 0.05). Compared with the period prior to the COVID-19 pandemic, the proportion of children in the PB group decreased (81 [60.90%] vs. 52 [39.10%]) whereas the proportion of children in the non-PB group increased (428 [41.31%] vs. 608 [58.69%]) during the pandemic (*P* < 0.0001) (Table 1).

**Table 1:** Demographic characteristics of children with MPP between the PB group and the non-PB group

Variables		Total	PB	non-PB	*P* value
Number		1169	133	1036	
**Sex (male), No. (%)**		632 (54.06)	71 (53.38)	561 (54.15)	1
**Age (mos), mean ± SD**		74.80 ± 30.64	80.40 ± 29.85	74.08 ± 30.68	0.3184
	age ≤ 36 mos, No. (%)	118 (10.09)	9 (6.77)	109 (10.52)	
	36 mos < age ≤ 60 mos, No. (%)	267 (22.84)	26 (19.55)	241 (23.26)	
	60 mos < age ≤ 84 mos, No. (%)	362 (30.97)	44 (33.08)	318 (30.69)	
	84 mos < age < 168 mos, No. (%)	422 (36.10)	54 (40.60)	368 (35.52)	
**Etiological diagnostic methods, No. (%)**					
	Single elevated immunoglobulin M	981 (83.92)	119 (93.23)	862 (83.20)	0.0931
	Seroconversion	979 (83.75)	120 (90.23)	859 (82.92)	0.0849
	PCR in BAL	1169 (100.00)	133 (100.00)	1036 (100.00)	1
**Incident season, No. (%)**					
	Spring (Mar, Apr, May)	111 (9.55)	18 (13.53)	93 (8.98)	0.4000
	Summer (Jun, Jul, Aug)	204 (17.45)	23 (17.29)	181 (17.47)	
	Fall (Sep, Oct, Nov)	537 (45.94)	60 (45.11)	477 (46.04)	
	Winter (Dec, Jan, Feb)	317 (27.12)	32 (24.06)	285 (27.51)	
**COVID-19 pandemic, No. (%)**					
	Before	509 (43.54)	81 (60.90)	428 (41.31)	< 0.0001^*^
	During	660 (56.46)	52 (39.10)	608 (58.69)	

Data are presented as mean ± SD and n (%). Differences between groups were determined by Kruskal–Wallis H test (mean ± SD) and Fisher’s exact test (proportions). ^*^P < 0.05

### Clinical manifestations

The median [IQR] duration of hospitalization was longer in the PB group than that in the non-PB group (9.00 [8.00, 10.00] days vs. 7.00 [6.00, 8.00] days; *P* < 0.0001). No differences were observed between groups for the time from illness onset to admission, duration of fever, and duration of cough. The incidence of lack of appetite was higher in the PB group than that in the non-PB group (98 [73.68%] vs. 616 [59.46%]; *P* = 0.0013). Compared with the non-PB group, patients in the PB group were more likely to have decreased breath sounds (93 [69.92%] vs. 607 [58.59%]; *P* = 0.0088). No remarkable differences in fatigue, dyspnea, wheezing, abdominal pain, diarrhea, chest pain, rales, rhonchi, tachypnea, hypoxia, and three depressions sign were observed between the two groups (Table 2).

**Table 2:** Clinical features and laboratory values of children with MPP between the PB group and the non-PB group

Characteristic		Total *N* = 1169	PB *N* = 133	non-PB *N* = 1036	χ²	*P* value
**Clinical data**						
	Time from illness onset to admission (days)	8.00 (6.00, 10.00)	7.00 (6.00, 9.00)	8.00 (6.00, 10.00)	1.7263	0.1889
	Duration of hospitalization (days)	7.00 (6.00, 9.00)	9.00 (8.00, 10.00)	7.00 (6.00, 8.00)	101.5953	< 0.0001^*^
	Duration of fever (days)	7.00 (5.00, 9.00)	7.00 (5.00, 8.00)	7.00 (5.00, 9.00)	0.0144	0.9046
	Duration of cough (days)	7.00 (5.00, 9.00)	7.00 (5.00, 9.00)	7.00 (5.00, 9.00)	1.0771	0.2993
	Fatigue	12 (1.03)	1 (0.75)	11 (1.06)	-	1
	Dyspnea	17 (1.45)	2 (1.50)	15 (1.45)		1
	Wheezing	46 (3.93)	4 (3.01)	42 (4.05)	-	0.8120
	Lack of appetite	714 (61.08)	98 (73.68)	616 (59.46)	-	0.0013^*^
	Abdominal pain	201 (17.19)	26 (19.55)	175 (16.89)	-	0.4653
	Diarrhea	75 (6.42)	10 (7.52)	65 (6.27)	-	0.5720
	Chest pain	33 (2.82)	3 (2.26)	30 (2.90)	-	1
**Physical sign**						
	Decreased breath sounds	700 (59.88)	93 (69.92)	607 (58.59)	-	0.0088^*^
	Rales	773 (66.12)	81 (60.90)	692 (66.80)	-	0.1461
	Rhonchi	334 (28.57)	35 (26.32)	299 (28.86)	-	0.5430
	Tachypnea	39 (3.34)	6 (4.51)	33 (3.19)	-	0.4387
	Hypoxia	40 (3.42)	6 (4.51)	34 (3.28)	-	0.4472
	Three depressions sign	20 (1.71)	4 (3.01)	16 (1.54)	-	0.2733
**Laboratory findings**						
	WBC (×10^9^/L)	8.12 (6.45, 10.22)	7.74 (6.29, 10.22)	8.15 (6.49, 10.24)	1.0187	0.3128
	N (×10^9^/L)	4.65 (3.43, 6.31)	5.12 (4.01, 7.00)	4.59 (3.36, 6.21)	8.9667	0.0027^*^
	L (×10^9^/L)	2.37 (1.67, 3.27)	1.70 (1.21, 2.40)	2.47 (1.79, 3.37)	58.0923	< 0.0001^*^
	PLT (×10^9^/L)	312.00 (252.00, 387.00)	288.50 (232.00, 336.00)	316.00 (256.00, 393.00)	16.3478	< 0.0001^*^
	CRP (mg/dL)	12.10 (4.60, 25.26)	26.74 (13.29, 52.31)	10.93 (3.97,22.73)	75.6621	< 0.0001^*^
	PCT (µg/L)	0.12 (0.07, 0.25)	0.25 (0.13, 0.44)	0.11 (0.07, 0.22)	46.2664	< 0.0001^*^
	ESR (mm/h)	25.00 (16.00, 40.00)	27.00 (20.00, 41.00)	24.00 (15.00, 39.00)	2.8851	0.0894
	D-dimer (mg/L)	0.64 (0.35, 1.31)	1.68 (1.04, 2.68)	0.58 (0.33, 1.09)	136.2348	< 0.0001^*^
	LDH (U/L)	324.00 (271.00, 409.00)	453.00 (349.00, 567.00)	315.00 (267.00, 388.00)	100.1390	< 0.0001^*^
	ALT (U/L)	13.00 (10.00, 20.60)	21.45 (14.80, 37.40)	12.60 (9.80, 19.00)	62.1271	< 0.0001^*^
	AST (U/L)	25.00 (20.00, 32.00)	33.00 (26.00, 50.00)	24.00 (20.00, 30.00)	69.6598	< 0.0001^*^
**CT scan**						
	Consolidation	921 (78.79)	102 (76.69)	819 (79.05)	-	0.5741
	Single lobar infiltrate	463 (39.61)	66 (49.62)	397 (38.32)	-	0.0187^*^
	Multilobar infiltrates (unilateral)	330 (28.23)	26 (19.55)	304 (29.34)	-	0.0252^*^
	Multilobar infiltrates (bilateral)	355 (30.37)	39 (29.32)	316 (30.50)	-	0.7657
	Pleural effusion	210 (17.96)	51 (38.35)	159 (15.35)	-	< 0.0001^*^
	Pericardial effusion	29 (2.48)	7 (5.26)	22 (2.12)	-	0.0391^*^
**Fiberoptic bronchoscopy**						
	Mucosal hyperemia and/or edema	711 (60.82)	32 (24.06)	679 (65.54)	-	< 0.0001^*^
	Mucosal erosion and/or necrosis	458 (39.18)	101 (75.94)	357 (34.46)	-	< 0.0001^*^
	Bronchial embolization	212 (18.14)	82 (61.65)	130 (12.55)	-	< 0.0001^*^
	Bronchial obstruction	14 (1.20)	2 (1.50)	12 (1.16)	-	0.6688
	Bronchiectasis	5 (0.43)	0 (0.00)	5 (0.48)	-	1

Data are presented as median [IQR] and *n* (%). Differences between groups were determined by Kruskal-Wallis H test (median [IQR]) and Fisher’s exact test (proportions). *P <0.05

### Laboratory tests

There were significant differences in median [IQR] levels for neutrophil (N), lymphocyte (L), platelet (PLT), C-reactive protein (CRP), procalcitonin (PCT), D-dimer, lactate dehydrogenase (LDH), alanine transaminase (ALT), and aspartate transaminase (AST) between the PB and non-PB groups (*P* < 0.05). The levels of N (5.12 [4.01, 7.00] vs. 4.59 [3.36, 6.21] ×10^9^/L; *P* = 0.0027), CRP (26.74 [13.29, 52.31] vs. 10.93 [3.97, 22.73] mg/dL; *P* < 0.0001), PCT (0.25 [0.13, 0.44] vs. 0.11 [0.07, 0.22] µg/L; *P* < 0.0001), D-dimer (1.68 [1.04, 2.68] vs. 0.58 [0.33, 1.09] mg/L; *P* < 0.0001), LDH (453.00 [349.00, 567.00] vs. 315.00 [267.00, 388.00] U/L; *P* < 0.0001), ALT (21.45 [14.80, 37.40] vs. 12.60 [9.80, 19.00] U/L; *P* < 0.0001), and AST (33.00 [26.00, 50.00] vs. 24.00 [20.00, 30.00] U/L; *P* < 0.0001) were significantly higher in the PB group than those in the non-PB group. Levels of L (1.70 [1.21, 2.40] vs. 2.47 [1.79, 3.37]×10^9^/L; *P* < 0.0001) and PLT (288.50 [232.00, 336.00] vs. 316.00 [256.00, 393.00]×10^9^/L; *P* < 0.0001) were significantly lower in the PB group than those in the non-PB group. There was no significant difference in the white blood cell count and erythrocyte sedimentation rate between groups (Table 2).

### CT scan results

The incidences of single lobar infiltrate (66 [49.62%] vs. 397 [38.32%]; *P* = 0.0187), pleural effusion (51 [38.35%] vs. 159 [15.35%]; *P* < 0.0001), and pericardial effusion (7 [5.26%] vs. 22 [2.12%]; *P* = 0.0391) were higher in the PB group than those in the non-PB group. The incidence of multilobar infiltrates (unilateral) (26 [19.55%] vs. 304 [29.34%]; *P* = 0.0252) was lower in the PB group than that in the non-PB group (*P* < 0.05). There were no significant differences in the incidence of consolidation and multilobar infiltrates (bilateral) between the groups (Table 2).

### FB findings and histopathological examination

**Fig. 2:**
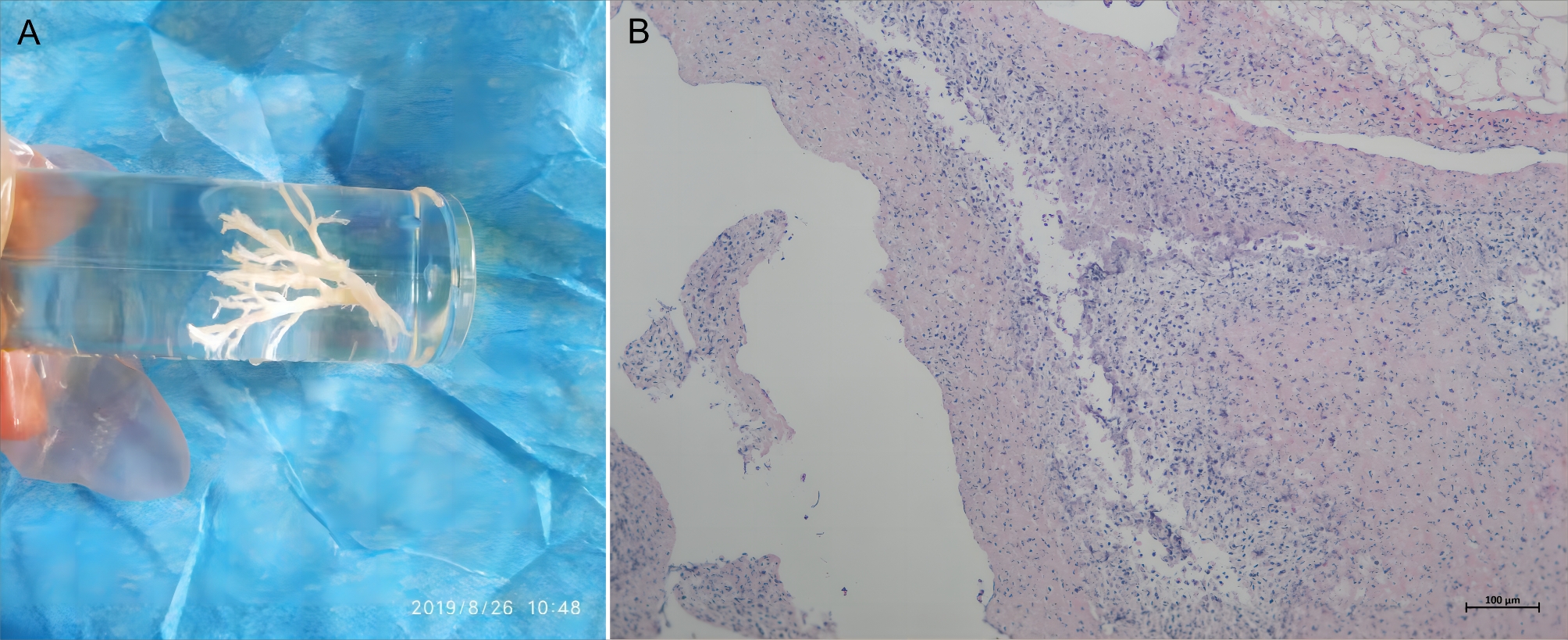
Bronchoscopic and histopathological examination findings. **A**: BC removed from one of patients in PB group; **B**: Hematoxylin and eosin examination of BC showed numerous inflammatory necrosis and neutrophils. Bar = 100 μm

The proportions of mucosal erosion and/or necrosis (101 [75.94%] vs. 357 [34.46%]; *P* < 0.0001) and bronchial embolization (82 [61.65%] vs. 130 [12.55%]; *P* < 0.0001) were higher in the PB group than those in the non-PB group, and the proportion of mucosal hyperemia and/or edema (32 [24.06%] vs. 679 [65.54%]; *P* < 0.0001) was lower in the PB group than that in the non-PB group. No remarkable differences in bronchial obstruction and bronchiectasis were observed between the two groups (Table 2). BC removed from one of patients in PB group is shown in Fig. 2A. Hematoxylin and eosin staining demonstrated that the BC contained numerous inflammatory necrosis and neutrophils (Fig. 2B).

### Multivariate regression analysis of PB in MPP

We performed multifactorial analysis with possible risk factors as the independent variables and development of PB as the dependent variable to determine the independent risk factors of PB in the context of MPP. The results showed that L levels (odds ratio [OR] = 1.591, 95% confidence interval [CI]: 1.236 ~ 2.096) and pleural effusion (OR = 2.466, 95% CI: 1.619 ~ 3.724) were independent factors influencing the development of PB with MPP (Table 3).

**Table 3:** Risk factors for PB in children with MPP

Variables	B	S.E.	Wald χ^2^	*P*	OR	95% CI
L (×10^9^/L)	0.4645	0.1348	11.8783	0.0006^*^	1.591	1.236 ~ 2.096
Pleural effusion	0.4514	0.1061	18.1149	< 0.0001^*^	2.466	1.619 ~ 3.724

^*^P < 0.05

## Discussion

PB is a relatively rare respiratory disease that can result in severe respiratory complications, such as respiratory failure and death [19, 22–24]. Its etiology is still poorly understood, but PB is associated with several cardiac and pulmonary conditions, including cyanotic congenital heart disease, asthma, cystic fibrosis, respiratory infections, lymphatic abnormalities, sickle cell anemia, neoplasms, and lung transplantation [25–28]. Respiratory infections are reported to be the main cause of PB in Asia, and MP is the primary pathogenic bacterium causing PB [12, 13, 29, 30]. In the present study, we analyzed clinical characteristics among children who had MPP with and without PB.

The underlying mechanisms of BC formation in patients with MPP are unclear at present. However, previous studies have shown that MP infection not only directly causes necrosis of the airway epithelium through adhesion damage and membrane fusion damage but also induces cilia removal dysfunction to promote the formation of mucus plug owing to excessive inflammation [12, 14, 31–33]. In this study, we found that the proportion of children with PB decreased dramatically during the COVID-19 pandemic. This may be owing to restrictive public health safety measures in place during the pandemic, such as wearing masks in public, sanitizing hands regularly, taking classes online, limiting public gatherings, health monitoring, travel restrictions, and border closures. These measures also helped to successfully control MP transmission [34–36].

We found that the duration of hospitalization in the PB group was longer than that in the non-PB group. This was consistent with reports by Zhong et al. [33] and Hua [37] and may be attributed to the following two aspects. On the one hand, the clinical manifestations of PB caused by MP infection are not specific, and MPP is considered self-limiting. PB caused by MPP cannot be treated in a timely manner during the early stage [38]. On the other hand, the mechanism of PB development after MP infection may be closely associated with MP drug resistance, increasing the difficulty of treatment [39].

The clinical manifestation of PB depends on the size and location of BCs and the primary disease. Our results showed that the incidence of lack of appetite was higher in the PB group than in the non-PB group. At present, there are no reports on the association between lack of appetite and PB. The main signs of MPP combined with PB are tachypnea, three depressions sign, and decreased breath sounds. Our study showed that patients in the PB group were more prone to decreased breath sounds, which is considered to be related to the positions and degrees of airway obstruction owing to BCs [13].

Laboratory tests are a convenient and practical method to assess the severity of PB. Levels of N, CRP, PCT, D-dimer, LDH, ALT, and AST were significantly higher in the PB group, and levels of L and PLT were significantly lower. These results were consistent with recent research [33, 40, 41]. The increase in N is related to the activation of neutrophils through toll-like receptor identifying MP lipid-associated membrane proteins after MP infection [42]. As sensitive indicators in the acute phase of inflammation, CRP and PCT are helpful in identifying the formation of PB caused by MPP [13]. LDH, a cytoplasmic enzyme that exists in various important organs, is a non-specific inflammatory biomarker of lysing in lung tissue or cell membrane damage. LDH serves as an important indicator to monitor infection severity and inflammatory disease [43]. The higher levels of N, CRP, PCT, and LDH in the PB group reflect an excessive inflammatory response, which can promote the formation of BCs. Systemic inflammation caused by MP infection leads to an imbalance between the blood coagulation and anticoagulation systems, causing a hypercoagulable state and higher D-dimer levels [14]. These lead to reduced air exchange capacity of the lung tissue and changes in the microcirculation, causing retention of inflammatory factors in the lungs, increased oozing of mucus, and the formation of BCs [13]. In addition to intrapulmonary damage, MP infection can cause extrapulmonary damage, such as damage to the liver [44]. Levels of ATL and AST reflect the degree of liver damage. The more severe the disease in children with MPP, the higher the levels of ALT and AST. In our study, the decreased level of PLT in the PB group was notable. As reported by Zhao et al. [41], the main reason for this is that MP infection and the formation of BCs could increase PLT damage and depletion.

Imaging findings in this study indicated that the incidence of single lobar infiltrate was higher and the incidence of multilobar infiltrates (unilateral) was lower in the PB group as compared with the non-PB group, suggesting that plastic casts in the PB group were more localized. Zhang et al. [42] found that PB caused by MP infection was likely to cause fragmented partial BCs with different pathogens. The incidences of pleural effusion and pericardial effusion were higher in the PB group than those in the non-PB group, which was consistent with data from studies in China and other countries suggesting that the local immune response was stronger in MP-induced PB cases than in non-PB cases [13, 37, 40–42, 45, 46].

In our study, FB and BAL were performed to make a definite diagnosis of PB and to clear airway obstructions. Under the bronchoscopy, the proportions of mucosal erosion and/or necrosis and bronchial embolization was higher in the PB group than those in the non-PB group, suggesting a strong inflammatory reaction; this was in line with the research findings of Zhang et al [42]. No remarkable difference in bronchial obstruction was observed between the two groups, we speculated might have been due to (1) BCs formation in segmental or subsegmental bronchus and (2) the timely intervention of bronchoscopy, preventing the spread of BCs throughout the airways [15].

Unlike previous single-factor analyses of risk factors for PB [33], in this study, we conducted multifactor analysis of the risk factors for developing PB in MPP. In line with published reports [37, 40–42], our results showed that L levels and pleural effusion were independent risk factors of PB with MPP. Our results will help clinicians to identify potential cases of PB for early intervention, especially FB, which is an invasive procedure.

Despite these strengths, several limitations remain in our study. First, we did not conduct patient follow-up. Second, this was a single-center study; further multicenter and large-sample studies are needed to reduce bias. Third, further exploration of the specific mechanism of BC formation caused by MPP is needed.

## Conclusions

In conclusion, children with PB caused by MPP had a strong and local inflammatory response. L levels and pleural effusion were independent risk factors of PB with MPP in children. Our findings will help clinicians to identify potential PB in pediatric patients for early and effective intervention.

## Data Availability

All data generated or analysed during this study are included in this published article.

## Abbreviations

MP: Mycoplasma pneumoniae
MPP: MP pneumonia
CAP: Community-acquired pneumonia
PB: Plastic bronchitis
BCs: Bronchial casts
FB: Fiberoptic bronchoscopy
BAL: Bronchoalveolar lavage
CT: Computed tomography
N: Neutrophil
L: Lymphocyte
PLT: Platelet
CRP: C-reactive protein
PCT: Procalcitonin
LDH: Lactate dehydrogenase
ALT: Alanine transaminase
AST: Aspartate transaminase
