# Publisher Correction: Clinical features and risk factors of plastic bronchitis caused by Mycoplasma pneumoniae pneumonia in children

**DOI:** 10.1186/s12890-023-02800-1

**Published:** 2023-12-11

**Authors:** Lei Yang, Yuyan Zhang, Changqing Shen, Zhouhua Lu, Tongshu Hou, Fenghai Niu, Yuzhong Wang, Jun Ning, Ruihan Liu

**Affiliations:** 1 Affiliated Hospital of Jining Medical University, Jining Medical University, Jining, 272000 Shandong China; 2 Postdoctoral Mobile Station of Shandong University of Traditional Chinese Medicine, Jinan, 250399 Shandong China; 3 The Second Clinical Medical College, Binzhou Medical University, Yantai, 264100 Shandong China

**Publisher Correction: BMC Pulm Med 23, 468 (2023)**

**https://doi.org/10.1186/s12890-023-02766-0**

Following publication of the original article [1], it came to the attention of the journal that due to a processing error, the order of the author list was incorrect. The author list has since been corrected and the corrected author list is reflected in this erratum. The publisher thanks you for reading this erratum and apologizes for any inconvenience caused.
